# The Screening and COnsensus Based on Practices and Evidence (SCOPE) Program Results of a Survey on Daily Practice Patterns for Patients with Metastatic Colorectal Cancer—A Swiss Perspective in the Context of an International Viewpoint

**DOI:** 10.3390/curroncol29080442

**Published:** 2022-08-06

**Authors:** Alexander R. Siebenhüner, Giorgia Lo Presti, Daniel Helbling, Petr Szturz, Christoforos Astaras, Yannick Buccella, Sara De Dosso

**Affiliations:** 1 Department of Medical Oncology and Hematology, Cantonal Hospital Schaffhausen, 8208 Schaffhausen, Switzerland; 2 Department of Internal Medicine, Ente Ospedaliero Cantonale, 6600 Locarno, Switzerland; 3 Gastrointestinales Tumorzentrum Zürich, 8032 Zurich, Switzerland; 4 Department of Oncology, University of Lausanne (UNIL) and Lausanne University Hospital (CHUV), 1015 Lausanne, Switzerland; 5 HUGE, Geneva University Hospitals, 1205 Geneva, Switzerland; 6 Stadtspital Zürich Triemli, 8063 Zurich, Switzerland; 7 Department of Medical Oncology, Oncology Institute of Southern Switzerland, Ente Ospedaliero Cantonale, 6500 Bellinzona, Switzerland; 8 Faculty of Biomedical Sciences, Università della Svizzera Italiana, 6900 Lugano, Switzerland

**Keywords:** Switzerland, metastatic colorectal cancer (mCRC), practice patterns, trifluridine-tipiracil, regorafenib, KRAS mutated mCRC, KRAS wildtype mCRC, sequential treatment

## Abstract

In Switzerland, physicians do not have national guidelines for metastatic colorectal cancer (mCRC) patient care and utilize international versions for management recommendations. Moreover, information about adherence to these guidelines and real-world practice patterns in Switzerland or other countries is lacking. The Screening and COnsensus based on Practices and Evidence (SCOPE) program were designed by an international expert panel of gastrointestinal oncologists to gather real-world insights in the current clinical setting to manage patients with mCRC who have received prior treatment. We sought to understand general practice patterns, the influence of molecular diagnostics (e.g., testing for KRAS, NRAS, BRAF, and MSI), tumor sidedness, and patient-centric factors on treatment selection utilizing in-person surveys and three hypothetical patient case scenarios. Here, we describe and evaluate the Swiss data from the SCOPE program within the context of an international viewpoint and discuss the findings of our analysis. In general, we find that the real-world clinical decisions of Swiss physicians (SWI) closely follow international (INT) recommendations and guidelines, largely paralleling their regional and international counterparts in using the two approved treatments in the third- and fourth-line settings, namely trifluridine-tipiracil and regorafenib. Finally, our data suggest a tendency toward the use of trifluridine-tipiracil (SWI: 79%; INT: 66%) over regorafenib (SWI: 18%; INT: 18%) as the preferred third-line treatment choice in mCRC patients regardless of KRAS status.

## 1. Introduction

Colorectal cancer (CRC) is the third most common type of cancer worldwide, accounting for 10.6% of all cancer cases [1]. In Switzerland, about 4500 new patients are diagnosed with CRC annually, corresponding to an age-standardized incidence rate of 46.5 and 29.6 cases per 100,000 European inhabitants in men and women, respectively, with the number of new cases growing every year [2]. Approximately 40% of those diagnosed with stages I-III become metastatic (mCRC) with an average estimated survival time of five to six months if left untreated [1,3,4,5]. On the contrary, treated mCRC shows a favorable five-year survival rate of 14% and a median survival time of 30 months [1,3,4,5]. According to the World Health Organization International Agency for Research on Cancer, in 2020, Switzerland had one of the most favorable colorectal mortality rates (per capita) in Europe [5]. This improvement is due, in part, to the development and application of large-scale clinical research studies on molecular diagnostics and precision therapeutics for advanced forms of colorectal cancer [2]. These strategies have improved the overall survival of mCRC patients in Switzerland from 19 months to more than three years over the last ten years [2].

Several national and international guidelines (e.g., German S3, European Society for Medical Oncology [ESMO], National Comprehensive Cancer Network [NCCN], etc.) now include similar recommendations for first-line (1L) and second-line (2L) treatment selection to guide physician decisions in daily practice [2,6,7,8]. However, guidelines beyond the second-line treatment lack a consensus on the optimized sequencing for the two agents approved for the third-line (3L) therapy in previously treated mCRC patients: Trifluridine/tipiracil (FTD/TPI) and regorafenib [1,2,6]. Once molecular characteristics and previous treatment regimens have been described, international guidelines recommend physicians define primary goals using therapy-related (e.g., toxicity, efficacy, route of administration) and patient-related factors (e.g., comorbidities, demographic characteristics, preferences) to optimize and select treatment options [2,9,10,11].

In Switzerland, physicians do not have a national guideline for mCRC patient care and utilize international versions for management recommendations [2]. Moreover, it is unknown whether physicians from Switzerland (and other countries) truly apply these recommendations to actual patients in a clinical practice setting [9]. In addition, as guidelines are based on the results of randomized controlled trials, there may be a gap between the efficacy reported in clinical trials and that observed in real-world daily practice, which may impact adherence [9,12].

The Screening and COnsensus based on Practices and Evidence (SCOPE) program was designed to gather real-world insights in current clinical to manage patients with mCRC who have received prior treatment [9]. The program was developed with a particular focus on delineating the different therapeutic options available, patients’ previous therapies, and understanding which criteria are considered important in treatment decision-making in real-world clinical practice [2,9,10,11,13].

In this study, we describe and evaluate the Swiss data from the SCOPE program within the context of the international viewpoint and discuss the findings of our analysis. This publication is focused on physicians working in Switzerland with the objective to (1) gather real-world insights on current clinical practices in the management of patients with mCRC who have received prior lines of palliative systemic treatment, (2) identify, describe, and analyze treatment disparities at local, national, and international levels, and (3) contribute to consensus decision-making on critical clinical questions for pretreated mCRC patients. Given high health expenditures in Switzerland, the near-universal health insurance coverage, and one of the highest life expectancies in the world, the relevance of describing influential factors on physicians’ treatment decisions is of high interest.

## 2. Materials and Methods

To understand more about physicians’ real-world patterns, an expert panel of gastrointestinal oncologists independently developed a survey to assess these practices. Only those physicians who were actively managing mCRC patients were eligible to participate. Physicians responded to questions relating to different hypothetical patient cases representing three treatment scenarios in the third- or fourth- lines and completed a declarative questionnaire section (Table S1). No patients were involved in this study, therefore, no ethics approval was required.

The expert panel of oncologists developed the three different hypothetical patient cases to be relevant to real-world practice. The expert oncologists first identified frequently encountered treatment scenarios within their practice that have posed challenges in the context of current treatment options. After reviewing and discussing each patient case, the expert panel refined their treatment histories to more closely model the therapeutic dosages and options seen in current clinical practice. The three patient cases are briefly described below, with a more detailed description (including each patient’s treatment history) found in Data S1.

- Case 1: A fit and active 54-year-old male with a left-sided, KRAS-wildtype colon adenocarcinoma
- Case 2: A 68-year-old female with a KRAS-mutated left-sided colon adenocarcinoma, comorbidities, and previous tolerability issues
- Case 3: An 82-year-old male with a KRAS-wildtype right-sided colon adenocarcinoma had comorbidities, limited support, and limited hospital accessibility

Data were collected from physicians at in-person meetings. Each of these cases was presented to the delegates by the meeting moderator in the respective countries (see Table S2 for a list of countries). Answers to the questions were collected via a Web application made available to each participant during the meetings, which enabled the anonymization of data to reduce any bias in responses. The Swiss (SWI) data were evaluated separately before being compared with the international (INT) viewpoint. Swiss data concerned only Swiss participants. However, international data included Switzerland as part of the total. We provided frequency tables and charts with percentages of answers from responders from the Swiss and international perspectives.

## 3. Results

### 3.1. Participant Demographics

Data were collected from 87 meetings conducted in 12 countries, with 706 participants. Data were collected between November 2018 and January 2020, from 87 meetings and 706 total participants. Only 629 physicians provided input on patient treatment within the final analysis, with baseline demographics summarized in Table S2. For both Switzerland and global participants, the majority were medical oncologists (SWI: 98%; INT: 69%), worked in university hospitals (SWI: 42%; INT: 47%), were seeing between 10–19 mCRC patients per month (SWI: 39%; INT: 31%), and were 35–55 years old (SWI: 68%; INT: 58%).

### 3.2. Practice Patterns

In an effort to understand real-world practices involving key clinical issues, physicians were questioned on: (a) Specific tests ordered for molecular diagnostics and (b) the impact of tumor sidedness on their first-line targeted therapy choice for patients with KRAS wildtype. To identify potential disparities of treatments at various geographical levels, (c) specific treatment goals in 1L vs. 3L mCRC patients and (d) patient case scenarios, as described earlier, were asked to participating physicians.

#### 3.2.1. Molecular Diagnostics

**Figure 1:**
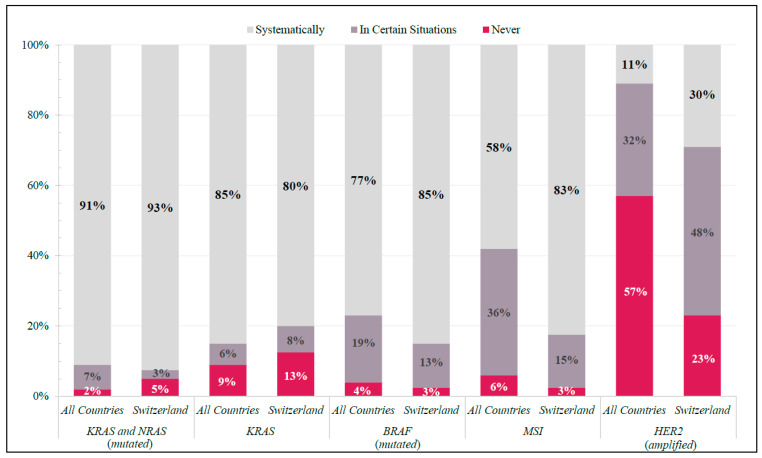
Molecular Diagnostics. Frequency of responses in Switzerland and All Countries (shown as percentage of total respondents in each group) to specific molecular diagnostic tests ordered in real-world clinical practice.

KRAS, NRAS, BRAF, and microsatellite instability (MSI) testing were systematically requested for mCRC patients by ≥80% of Swiss physicians, whereas HER2 was not (30%) (Figure 1). Internationally, KRAS and NRAS and BRAF testing were systematically requested by ≥91% and ≥77%, respectively. MSI and HER2 tests were not systematically requested among all global participants (MSI: ≤58%; HER2: ≤11%) (Figure 1).

**Figure 2:**
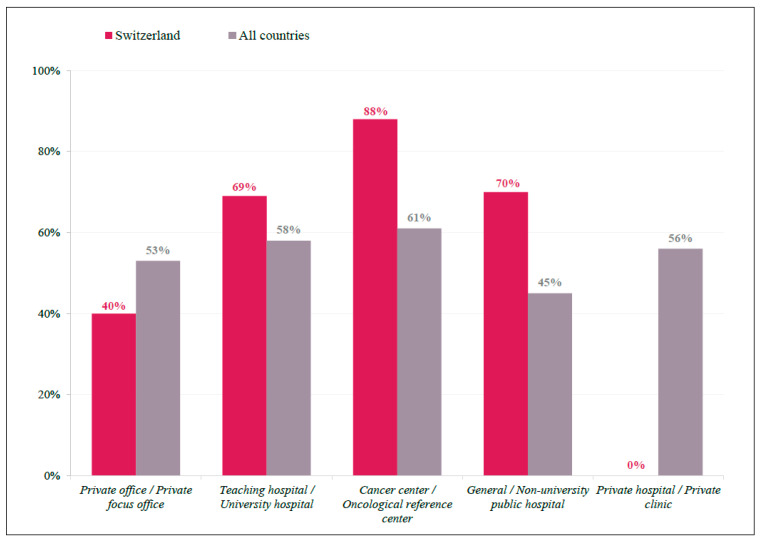
Tumor Sidedness by Participant Location. Bar graph representing practice demographics of respondents indicate tumor sidedness as having a ‘high impact’ on treatment decision, displayed as a percentage of total respondents for Swiss (red) and International (grey) participants.

There were seven countries where >2/3 of physicians responded on tumor sidedness as having a high impact on clinical decision making: Austria, Switzerland, Croatia, Hungary, Italy, Argentina, and Germany. In Switzerland, 70% of physicians indicated tumor sidedness as a factor with a high impact, which was approximately 14% higher than that observed in all other countries (56%) (Figure S1). In addition, tumor-sidedness seemed to be more important for Swiss physicians working in cancer centers (88%) than in general/university hospitals (70%), and an even lower preference for this factor was seen among international respondents in general/university hospitals (45%) (Figure 2).

#### 3.2.2. Treatment Goals

**Figure 3:**
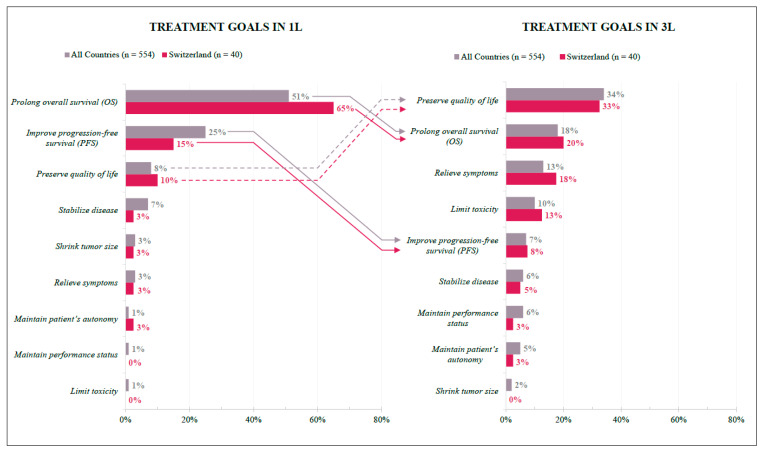
Treatment Goals in 1L and 3L. Preferences of Swiss (red) and International (grey) respondents expressed as percentages according to their answers. Arrows indicate change in 1L and 3L preferences; solid lines = negative change in relative (1L to 3L) position ranking; dashed lines = positive change in relative (1L to 3L) position ranking.

In Switzerland, physicians’ treatment goals differed between the first and third lines but were overall homogenous with international findings. For the 1L treatment, efficacy is the primary goal: Prolonging overall survival was ranked as the top priority by most respondents (SWI 65%; INT 51%), followed by improving progression-free survival (SWI 15%; INT 25%). In 3L, one-third of respondents listed quality of life results as the primary goal (SWI 33%; INT 34%), followed by prolonged overall survival (SWI 20%; INT 18%) and relieving symptoms (SWI 18%; INT 13%) (Figure 3).

#### 3.2.3. Patient-Centric Factors Influencing Treatment Choice in 3L and Beyond

In a 3L setting, patient-centric factors were found to contribute to the choice of treatment route of administration (oral vs. IV) in Swiss physicians, including the patient’s general condition, proximity to the hospital, and age (Figure S2). In general, this reflected international findings, however, Swiss physicians tended to be more influenced by patients’ limited knowledge, low caregiver support, and activity levels compared to international response rates.

### 3.3. Patient Cases

Case 1: A fit and active 54-year-old male with a left-sided, KRAS-wildtype colon adenocarcinoma

**Figure 4:**
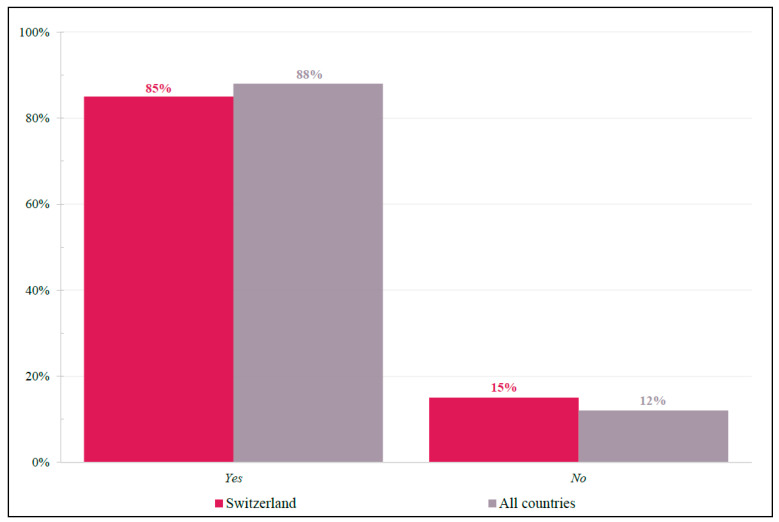
Case 1: Trifluridine-tipiracil Appropriateness. Yes/No response frequencies of physicians when asked whether they considered trifluridine-tipiracil as an appropriate 3L treatment choice for patient case #1. Shown as a percentage of respondents.

For this fit and active patient, physicians were asked whether trifluridine-tipiracil was considered an appropriate third-line treatment choice who received anti-epidermal growth factor receptor (anti-EGFR) and anti-vascular endothelial growth factors (anti-VEGF) as first-line and second-line treatments, respectively (Figure 4). Swiss responses (Yes = 85%; No = 15%) closely resembled international findings (Yes = 88%; No = 12%).

When asked to specify from a list of factors what drove their treatment choice, the majority of Swiss (60%) and international physicians (54%) selected “survival data in the RECOURSE trial” and “because of safety profile” (SWI: 45%; INT: 44%) (Figure S3). International respondents, however, also indicated “Disease control rate in the RECOURSE trial” (44%) and “[It has an] oral route of administration” (47%) as other highly impactful factors.

In 4L after FTD/TPI, Swiss physicians preferred regorafenib over “rechallenge with anti-EGFR” and “enroll in clinical trial” with preference rates of 41%, 28%, and 15%, respectively (Figure S4). Internationally, regorafenib and “enroll in clinical trial” were more closely ranked (31% and 29%, respectfully), with a rechallenge being selected in 19% of responses. These results were consistent with the respective national and international guidelines.

Case 2: A 68-year-old female with a KRAS-mutant left-sided colon adenocarcinoma, comorbidities, and previous chemotherapy tolerability issues; never exposed to FTD/TPI

When asked about their preferred 3L to 4L treatment for this patient, the use of FTD/TPI (SWI: 79%; INT: 66%) was the preferred 3L treatment choice over regorafenib (SWI: 18%; INT: 18%) (Figure S5). When still considering respondents’ 3L treatment choice, regorafenib was the preferred treatment choice (SWI: 68%; INT: 55%) over FTD/TPI (SWI: 14%; INT: 17%) in the 4L setting (Figure S6). When examining preferred 3L to 4L treatment options in sequence, the majority of respondents preferred FTD/TPI to regorafenib, respectively (SWI: 68%; INT: 54.3%), compared to regorafenib to FTD/TPI (SWI: 14%; INT: 15.2%), respectively (Figure S7).

Case 3: An 82-year-old male with a KRAS-wildtype right-sided colon adenocarcinoma who has comorbidities, limited support, and difficult hospital accessibility

**Figure 5:**
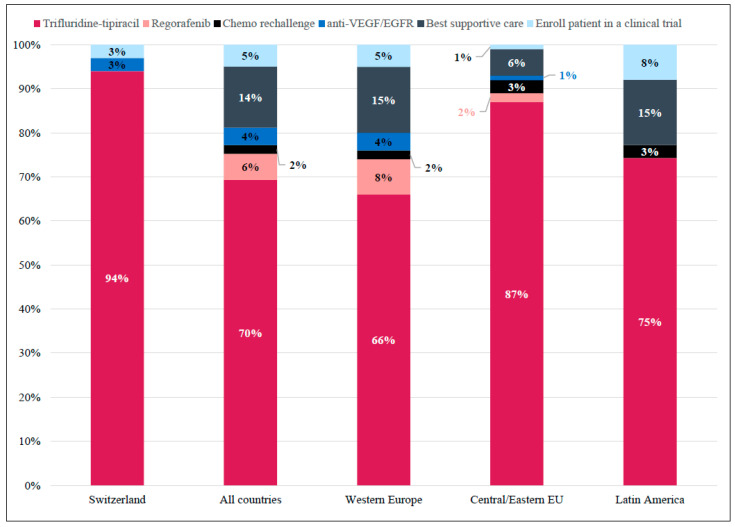
Case 3: Preferred Treatment Choice in the Third-line per Region. Responses for whether trifluridine-tipiracil would be considered a suitable third-line treatment option for a patient with comorbidities, limited support, and difficult hospital accessibility. Shown as percentage of total responses in Switzerland (n = 36), All countries (Western Europe + Central/Easter Eu + Latin America; N = 529), Western Europe (n = 421), Central/Eastern Europe (n = 68), and Latin America (n = 40).

Most Swiss physicians (94%) considered FTD/TPI the most appropriate treatment in 3L for patients with comorbidities, limited support, and difficult hospital accessibility (Figure 5).

Internationally, 70% of respondents agreed with this treatment as being appropriate. This response was primarily homogeneous across countries, except for Germany, the United Kingdom, and Spain, where best supportive care was also considered appropriate (Figure S8).

## 4. Discussion

The purpose of this publication is to describe, compare, and contrast factors that contribute to 3L or 4L treatment decisions of physicians in Switzerland who treat mCRC patients in their daily practice. Our data reveals that Swiss physicians generally follow the international recommendations (e.g., NCCN and ESMO guidelines) in real-world clinical practice when managing patients with mCRC, reflecting similar findings from the previously reported global SCOPE publication [9]. In that report, Swiss physicians’ drivers of 3L/4L treatment choices reflected an international viewpoint and tended to follow the overall trend of other countries closely. Practice patterns revealed by this survey indicate that Swiss physicians generally follow the recommendations of several international guidelines (NCCN, German S3, ESMO) on the use of the two approved treatments in the 3L/4L settings [6,7,8].

Treatment goals varied according to treatment line in both analyzed groups (Swiss and international), with >50% of respondents (SWI: 51%; INT: 65%) selecting “preserving overall survival” being the primary goal in the 1L setting, while in 3L >30% opted for “preserving quality of life.” Interestingly, “preserving quality of life” was ranked third, with ≤10% of respondents from both groups selecting this option as the primary 1L treatment goal. There appears to be a consensus on 1L treatment goals between Swiss and international respondents, with both focusing on efficacy outcomes as opposed to the less agreed upon 3L treatment goal, which took patient factors (i.e., preserving quality of life) more into consideration. Given the ranked order of 1L and 3L treatment goals are aligned for both Swiss and international participants, the improved mortality rates seen in late-stage colon cancer in Switzerland suggest differences in implemented treatment regimens may depend on patient-centric factors rather than provider-related decisions. Indeed, patient-centric factors and preferences become more important to Swiss physicians in later (≥3L) treatment lines, with proximity to the hospital, patient’s general condition, and age exerting a major influence on the route of administration (oral vs. intravenous). Moreover, in contrast to their international counterparts, Swiss physicians were more likely to consider an intravenous route of administration in patients with limited understanding of treatment options and less likely to consider an oral route of administration if patients had low caregiver support.

In evaluating current practice patterns, Swiss physicians tended to consider tumor characteristics more frequently than their international peers. While KRAS, NRAS, and BRAF testing were frequently requested in all countries, Swiss physicians also included MSI testing when deciding treatment options as part of their systematic process. When asked about tumor sidedness, Switzerland was one of seven countries where >2/3 of physicians considered it a “high-impact” feature, with many of these seven countries also reporting higher rates of systematic MSI testing. However, this overlap was not complete, and such variability may be a result of limited access to proper diagnostic equipment or subsequent treatment options. Interestingly, these results align with a 2018 study designed to capture 1L treatment patterns of mCRC patients in real-world clinical practices in five European countries (France, Germany, Italy, Spain, and the United Kingdom) [14]. In that study, despite tumor-sidedness and biomarker status being the main drivers of (1L) treatment selection, they were not consistently incorporated into mCRC patient regimens. The finally adopted treatment strategies varied considerably between countries, testifying reimbursement issues as one of the primary barriers [14]. Thus, the variations in decision-making showed in our study may be explained by reimbursement and other health system socioeconomic issues, which is also supported by other studies [15,16]. Improved access for Swiss physicians to approved treatment options for patients with deficient mismatch repair/microsatellite instability (MSI)-high tumors, including pembrolizumab or nivolumab and ipilimumab, may be a driving factor for these differences, though further data collection is needed.

The latest ESMO and NCCN guidelines consider FTD/TPI and regorafenib as equal recommendations for 3L/4L treatment options in mCRC patients experiencing disease progression after two lines of standard therapy but still highlight the better safety profile of FTD/TPI [3,7,12,13]. Nonetheless, deciding the optimal treatment sequence is a key clinical question currently faced by practicing physicians [9,11]. In the pivotal phase III RECOURSE study (NCT01607957), FTD/TPI significantly improved overall survival and progression-free survival versus placebo in patients with mCRC who had progressed on standard therapies [17]. However, quality of life was not assessed in this study [18]. Our data show a major consensus among Swiss physicians with FTD/TPI as the preferred 3L treatment option, regardless of comorbidities or prior intolerance, and consider the RECOURSE survival data as a significant driving factor. Responses of Swiss physicians reflect similar findings on the international level, but the oral route of administration has less influence on their decisions. In KRAS-WT patients with comorbidities, limited support, and difficult hospital access, Swiss physicians were less likely to consider any other treatment options when compared with their international or regional colleagues, with 94% of Swiss respondents indicating FTD/TPI is the most appropriate treatment.

When examined at the country level, Swiss response frequencies and distribution in the context of preferred 3L or 4L treatment options closely resembled those of other countries, except for Germany, which shows considerable differences in treatment-related question types. These differences may be explained by recent reassessments within the German S3 guidelines, where the ranking of regorafenib was replaced with a “weak” (3L) recommendation compared to FTD/TPI (“moderate”) and rechallenge (“moderate”) options [2,8]. As a result, regorafenib is no longer available for reimbursement in Germany, bringing further evidence to the presumption that national health system economics plays an important role in treatment decision-making [2,8,19]. In Switzerland, the lack of national guidelines may reduce treatment selection bias, as the physicians can utilize data from clinical trials at their discretion for the benefit of their patients. This may be supported by findings of a 2018 survey among United States oncologists on adherence to new NCCN universal screening recommendations of MSI in mCRC patients, where the primary reason for the adoption of the new standard was that “it allows for better patient management” (82.8%) followed by “it is standard practice” (70.2%) [20].

Preferred 4L treatment options for Swiss physicians tended to be more variable than their 3L preferences in terms of response frequencies and distribution. Our data suggest that Swiss physicians prefer FTD/TPI followed by regorafenib as the optimal treatment sequence in patients with comorbidities and previous tolerability issues, regardless of KRAS mutation status. However, after regorafenib, Swiss physicians preferred “rechallenge” as the next most appropriate 4L option and were less likely to enroll patients in a clinical trial when compared with their international counterparts. The reasons for this finding are unclear and may stem from lower rates of clinical trials throughout Switzerland or increased availability of other viable therapeutic options for >3L (FTD/TPI) in mCRC patients. In other countries, clinical trials may offer cancer patients a solution with low-cost sharing, enabling them to access potentially life-saving therapeutics. However, Swiss physicians and the country’s residents might not face this barrier as frequently due to universal health coverage and may find their current therapeutic options preferable to enrollment in clinical trials.

## 5. Conclusions

The SCOPE international program has provided enhanced insights into real-world treatment practices for patients with mCRC, highlighting how logistic, medical, geographic, and economic factors may contribute to a physician’s treatment decision. Our analysis of the Switzerland dataset demonstrated that efficacy and quality of life are the two most important goals in the 3L setting for Swiss physicians. This is reflected by the preferred 3L and 4L treatment choices for patients with mCRC. In addition, this survey revealed that the daily practice patterns of Swiss physicians reflect recommendations of several international guidelines on using the two approved treatment regimens in the 3L and 4L settings. Finally, our data suggest a tendency toward the use of FTD/TPI followed by regorafenib in mCRC patients regardless of KRAS status. As new treatment options and research results become available, it is important to follow real-world practice trends in similar studies both in Switzerland and internationally. Currently, a second SCOPE GI program is collecting data in 2022.

## Data Availability

The data presented in this study are available on request from the corresponding author. The data are not available in a public repository due to their non-clinical nature.

## Supplementary Materials

The following supporting information can be downloaded at: https://www.mdpi.com/article/10.3390/curroncol29080442/s1, Table S1: Declarative questions of the survey; Table S2: Demographics of participants; Data S1: Disease characteristics and treatment history of the three patient cases; Figure S1: Impact of Tumor Sidedness by Location; Figure S2: Patient-centric Factors Influencing Physician’s Treatment Choice.; Figure S3: Case 1: Treatment Choice Drivers.; Figure S4: Treatment Choice In 4L Post-Trifluridine-Tipiracil.; Figure S5: Case 2: Preferred 3L Treatment Option; Figure S6: Case 2: Preferred Treatment Choice in 4L (post-regorafenib).; Figure S7: Combined 3L to 4L Preferred Treatment Options Shown in Sequence; Figure S8: Case 3: Preferred Treatment Choice as 3L per Country.
