# Supplementary material for: The Screening and COnsensus Based on Practices and Evidence (SCOPE) Program Results of a Survey on Daily Practice Patterns for Patients with Metastatic Colorectal Cancer—A Swiss Perspective in the Context of an International Viewpoint
Source: Curr Oncol. 2022 Aug 6;29(8):5604–15. doi: 10.3390/curroncol29080442 (PMC9406863; doi:10.3390/curroncol29080442)
Supplement: Supplementary file 1 [file curroncol-29-00442-s001.zip › Figures S1-S8.pdf]

Supplementary Data, S2: *Additional figures referred to in text.*

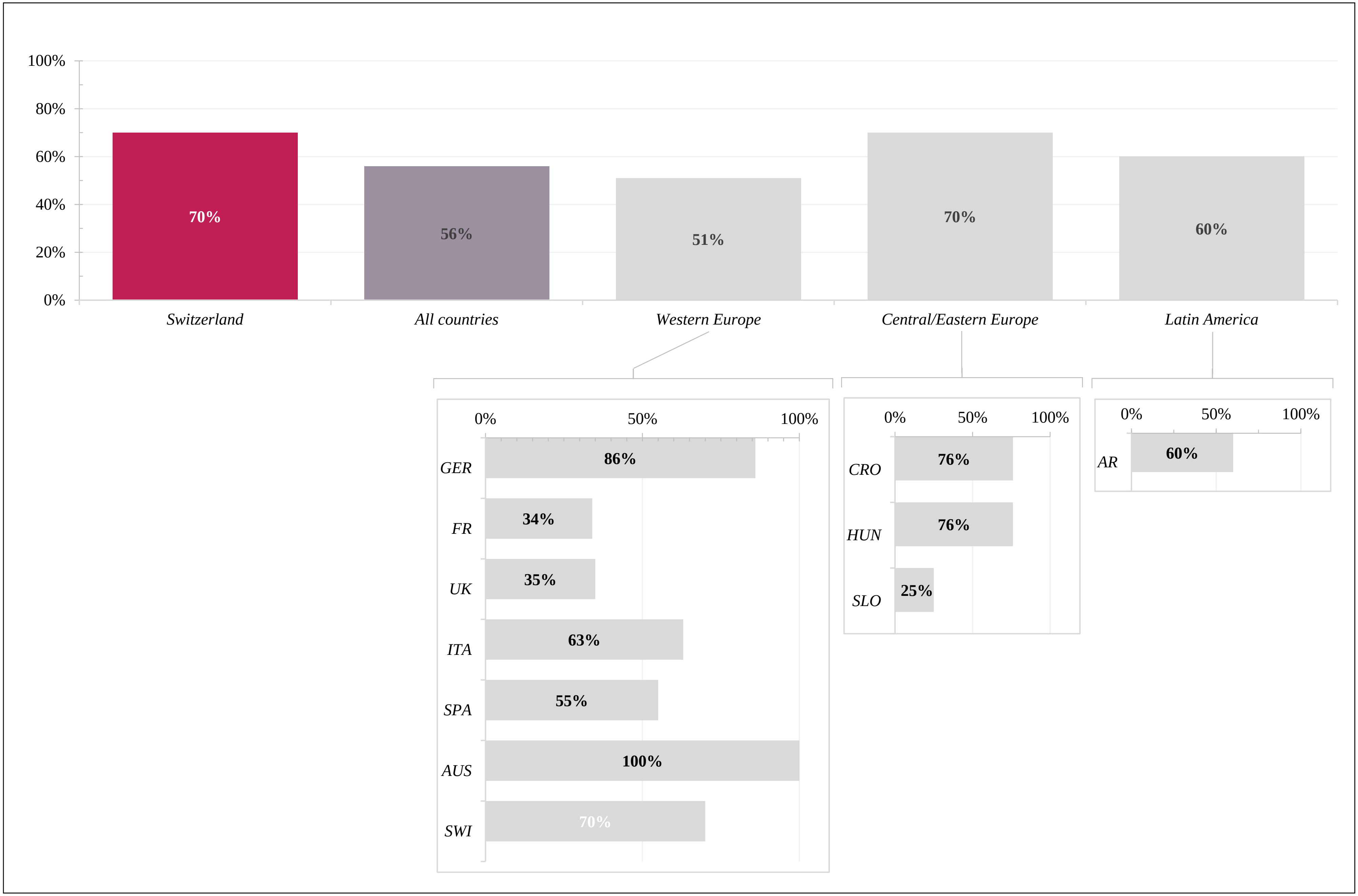

Supplementary Figure S1. **Impact of Tumor Sidedness by Location**. The impact of tumor sidedness on 1L treatment choice for RAS WT patients in all participants' countries, different regions (Western/Central/Eastern Europe and Latin America), and their constituent countries, including Switzerland.

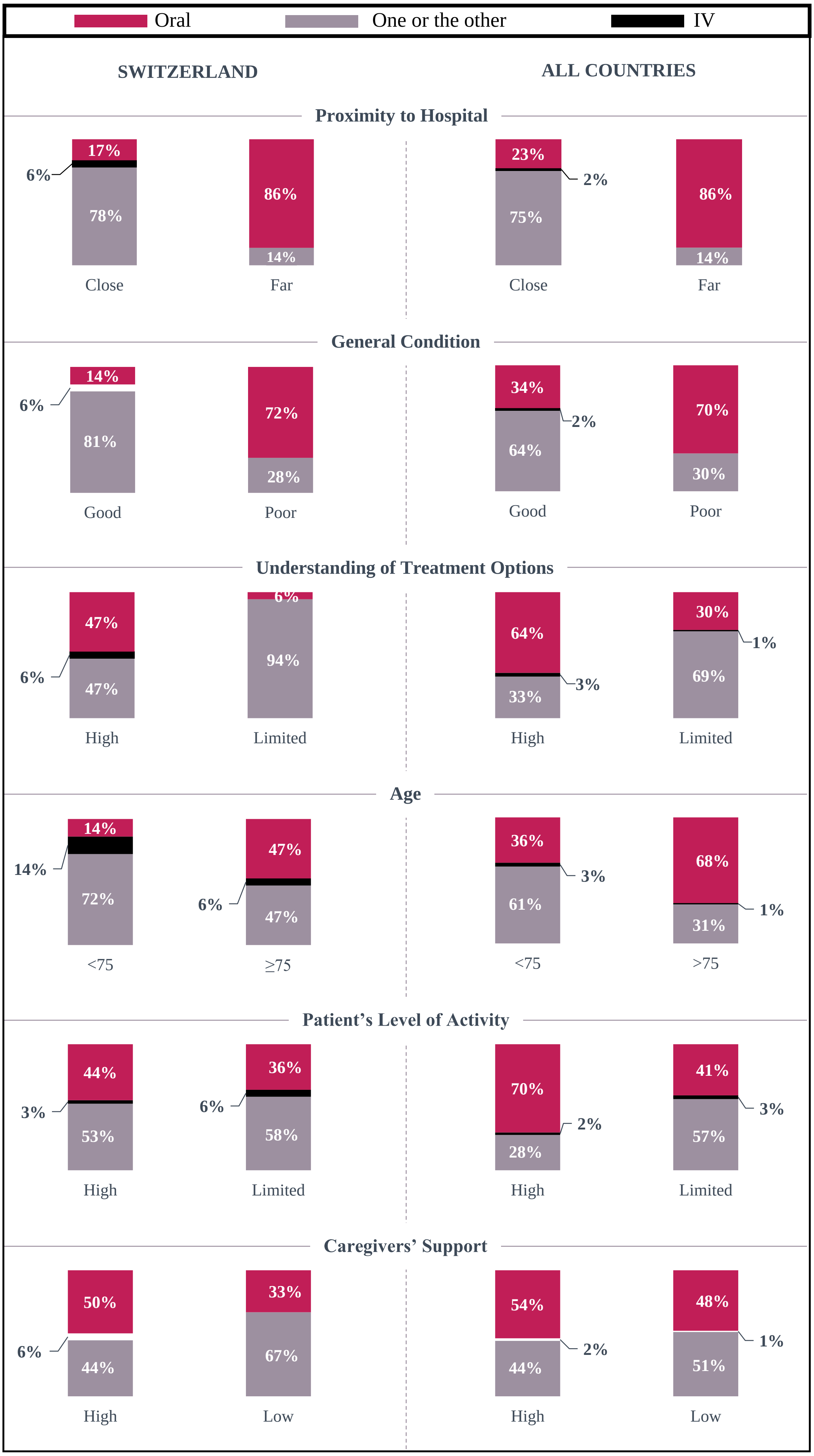

Supplementary Figure S2. Patient-centric Factors Influencing Physician's Treatment Choice. The bar chart shows percentage of Swiss (on the left side) and international (on the right side) responses to questions about which route of administration would be favored in the third-line treatment setting (either intravenous, oral, or no preference) on the basis of patient-related factors, including proximity to hospital (close vs far), patient's general condition (good vs poor), understanding of treatment options (high vs limited), age (<75 vs ≥75), patient's level of activity (high vs limited), and caregiver support (high vs low).

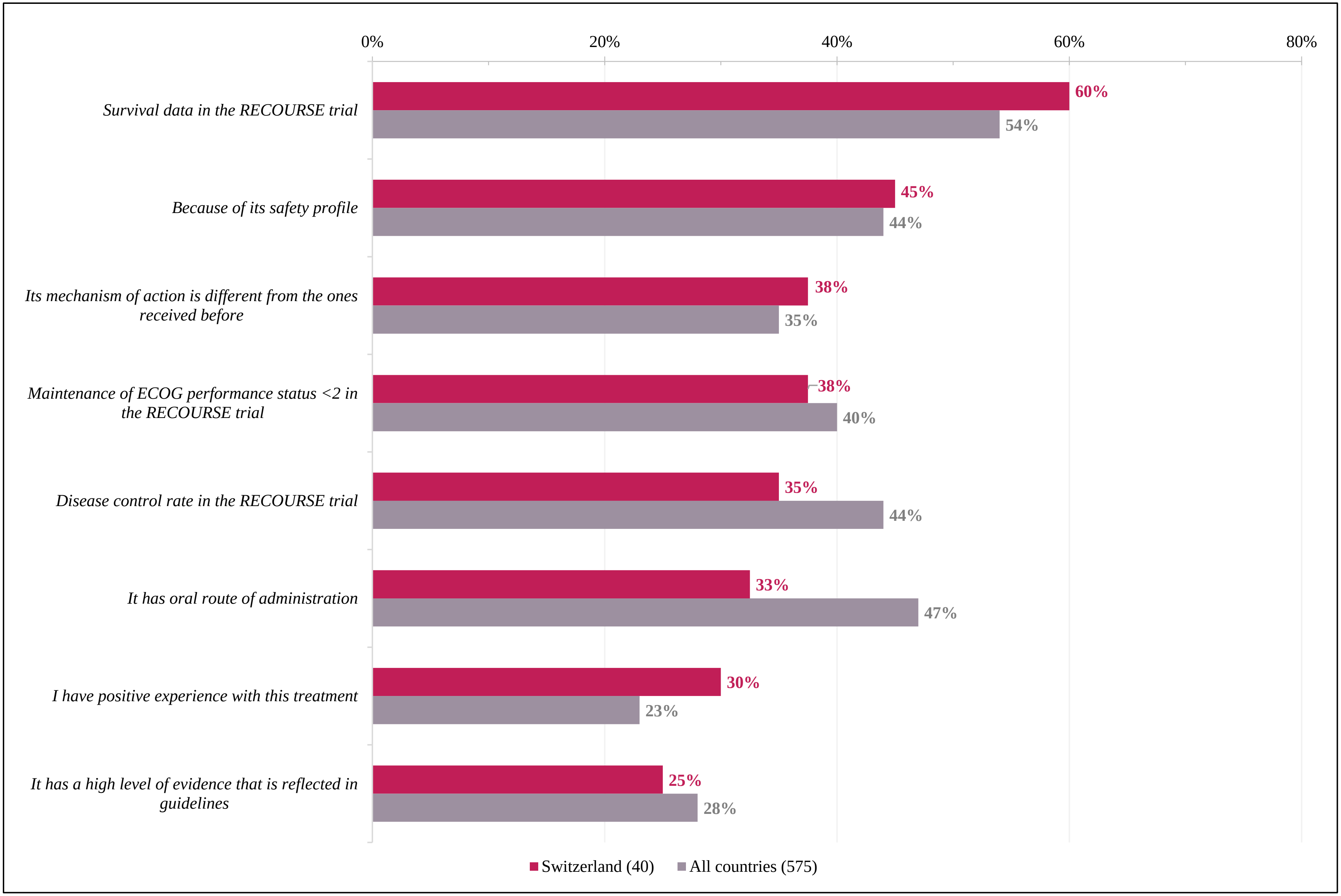

Supplementary Figure S3. Case 1: Treatment Choice Drivers. Response frequency to the appropriateness of trifluridine-tipiracil treatment in 3L for fit and active patients who received anti-EGFR in 1L and anti-VEGF in 2L in Swiss (red) and international (grey) participants. Shown as percentage of respondents.

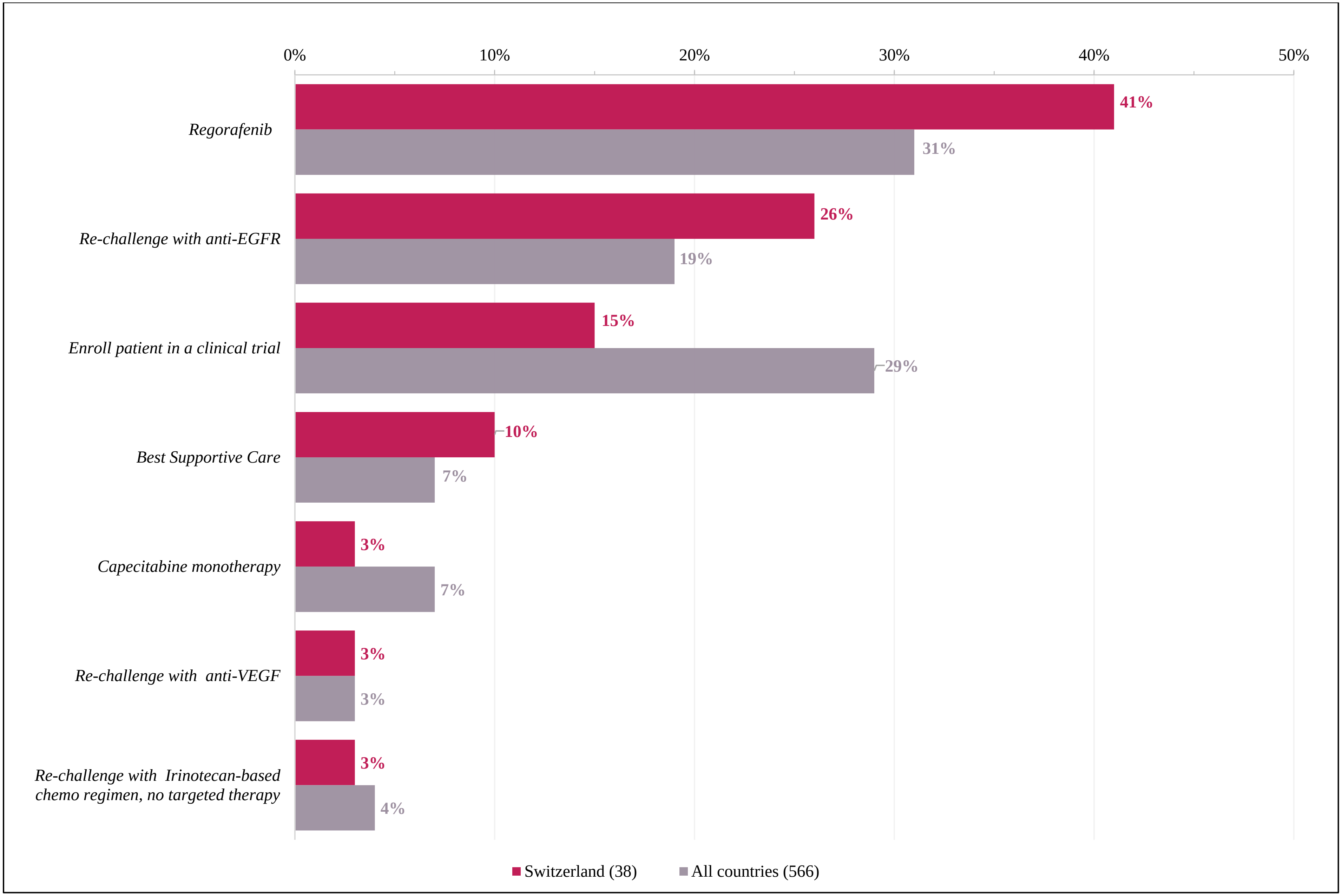

Supplementary Figure S4. Treatment Choice In 4L Post-Trifluridine-Tipiracil. Responses for preferred treatment for 4L for KRAS-WT, fit, and active patient without comorbidities or previous tolerability issues among Swiss and international participants. Shown as percentage of respondents.

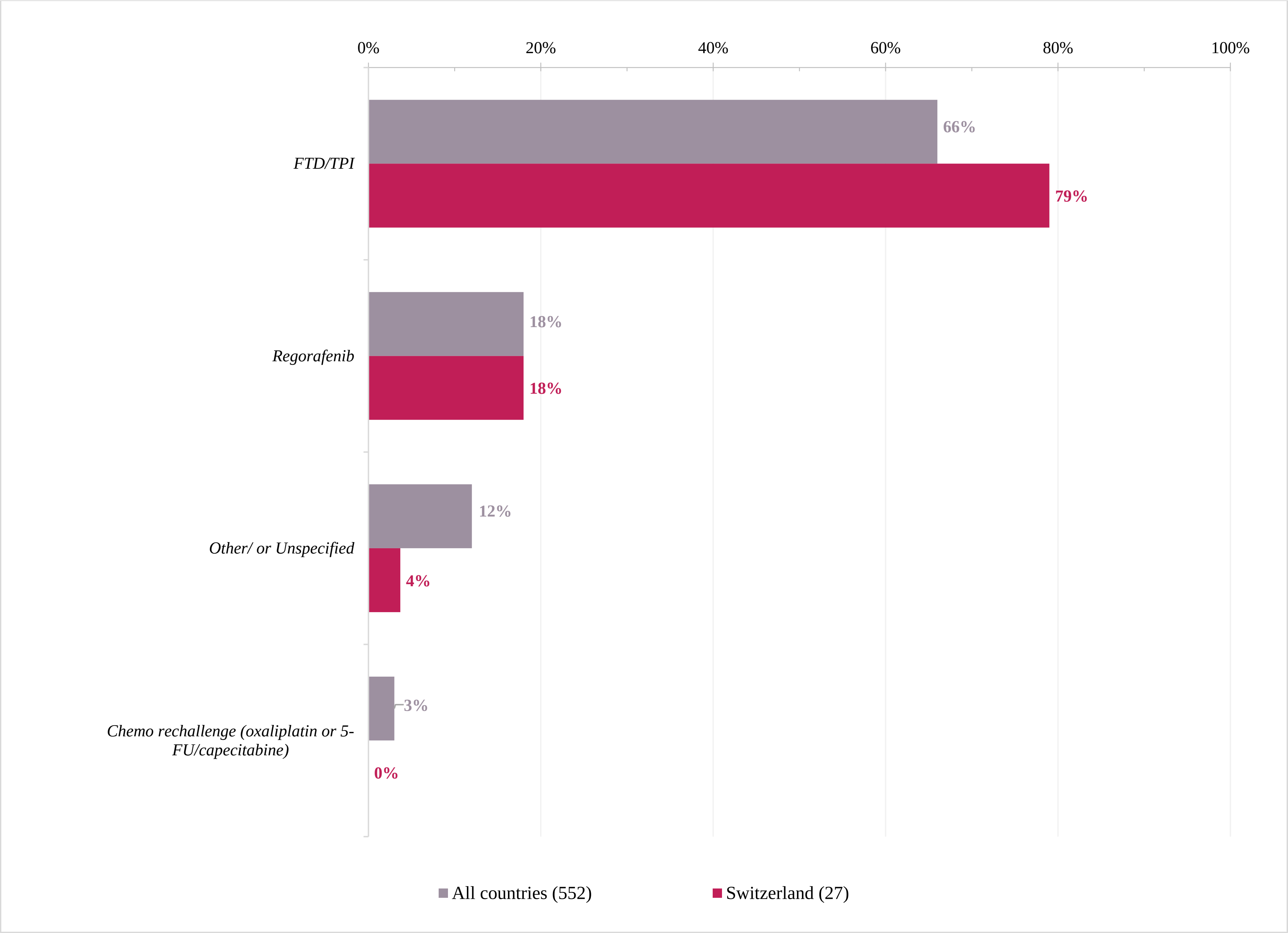

Supplementary Figure S5. Case 2: Preferred 3L Treatment Option. Bar graphs demonstrate the percentage of responses for Swiss (red) and international (grey) participants when asked about the preferred treatment option for these patients in the third-line setting.

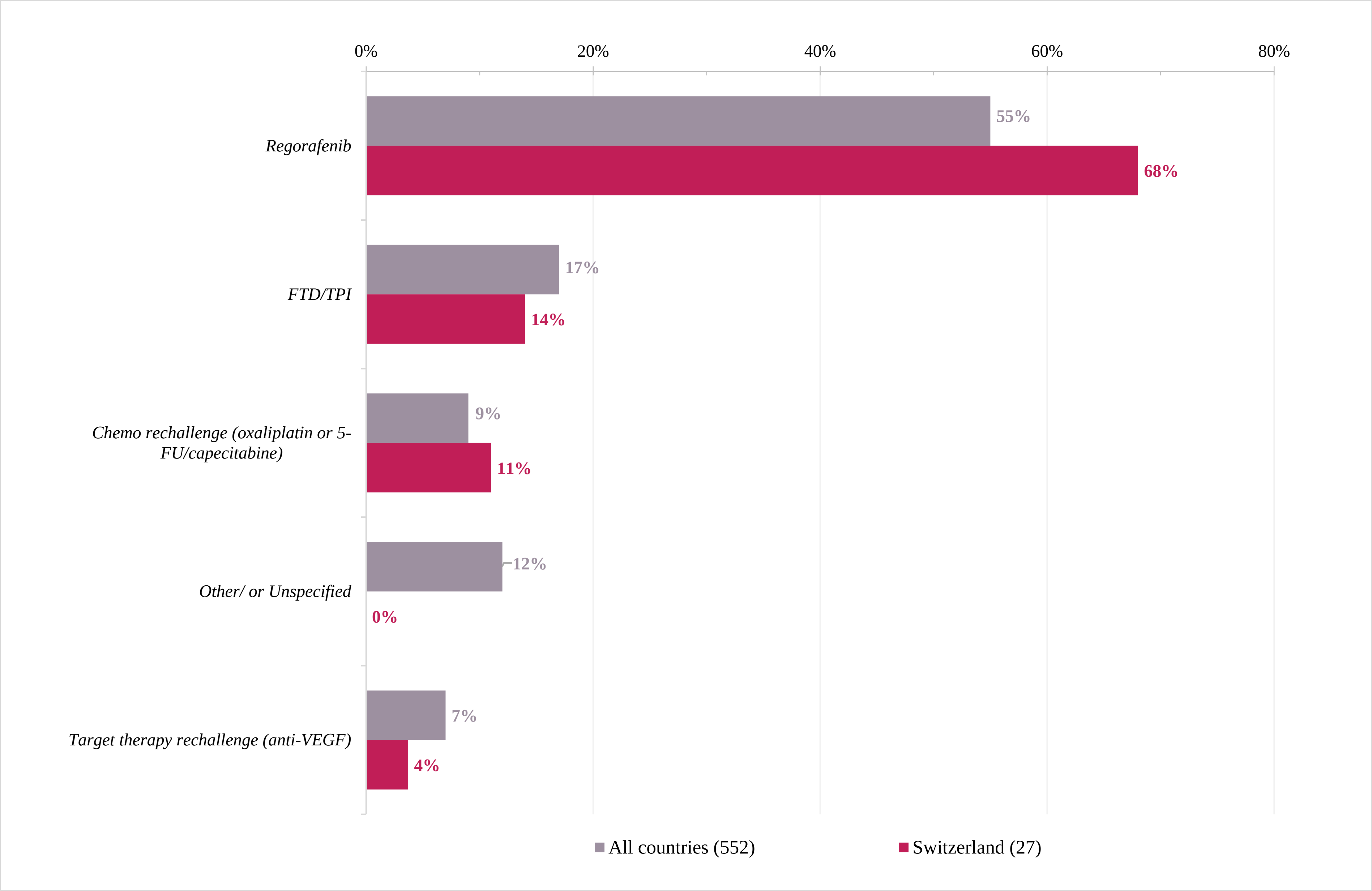

Supplementary Figure S6. Case 2: Preferred Treatment Choice in 4L (post-regorafenib). Bar graphs demonstrate the percentage of responses for Swiss (red) and international (grey) participants when asked about the preferred treatment option for these patients in the fourth line setting when regorafenib was used in the third line.

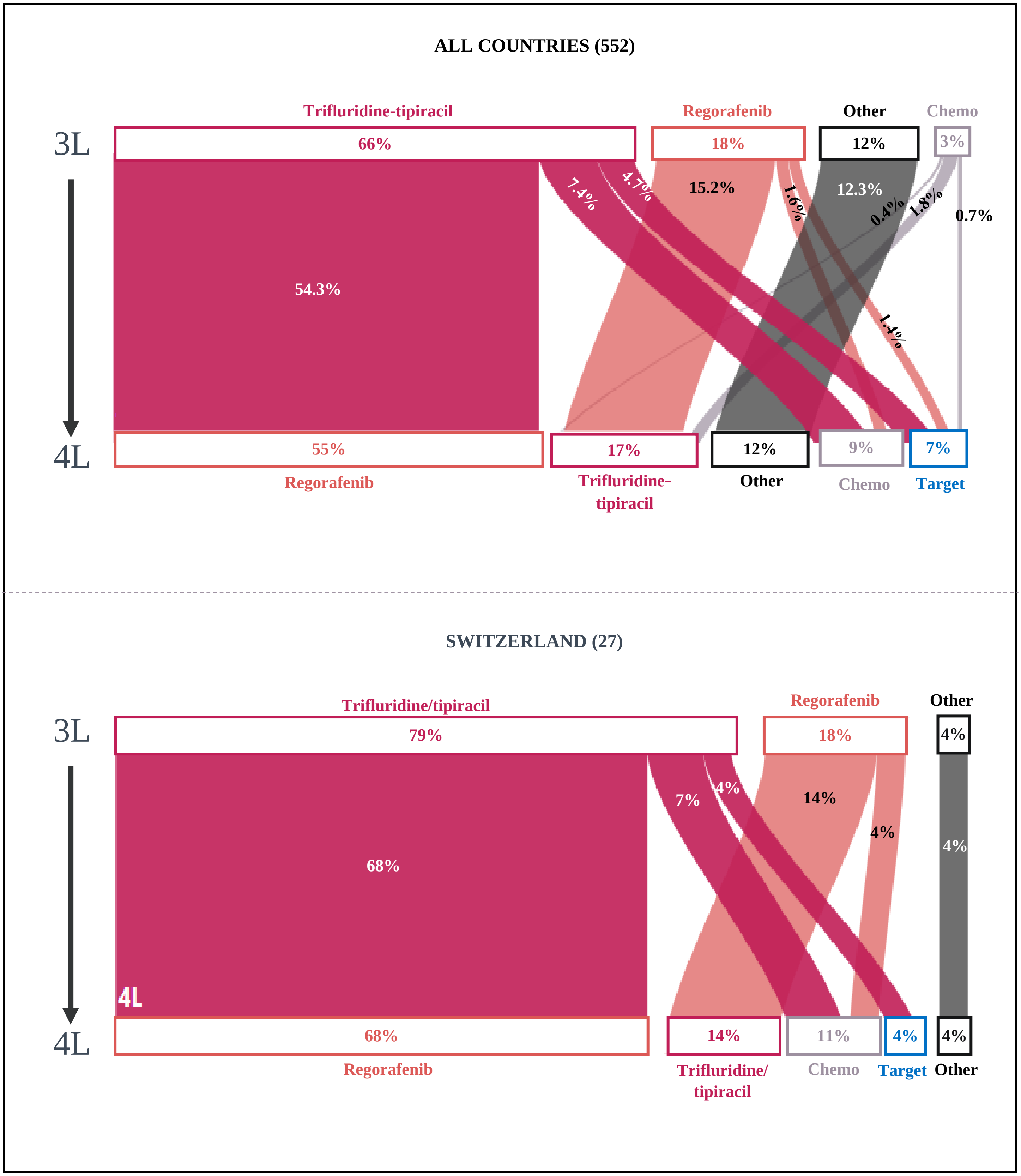

Supplementary Figure S7. Combined 3L to 4L Preferred Treatment Options Shown in Sequence. Bar graphs from Figures 8a and 8b were combined to demonstrate the percentage of respondents from all countries (above the dotted line) and Switzerland (below the dotted line) and the preferred sequence of 3L and 4L treatment options.

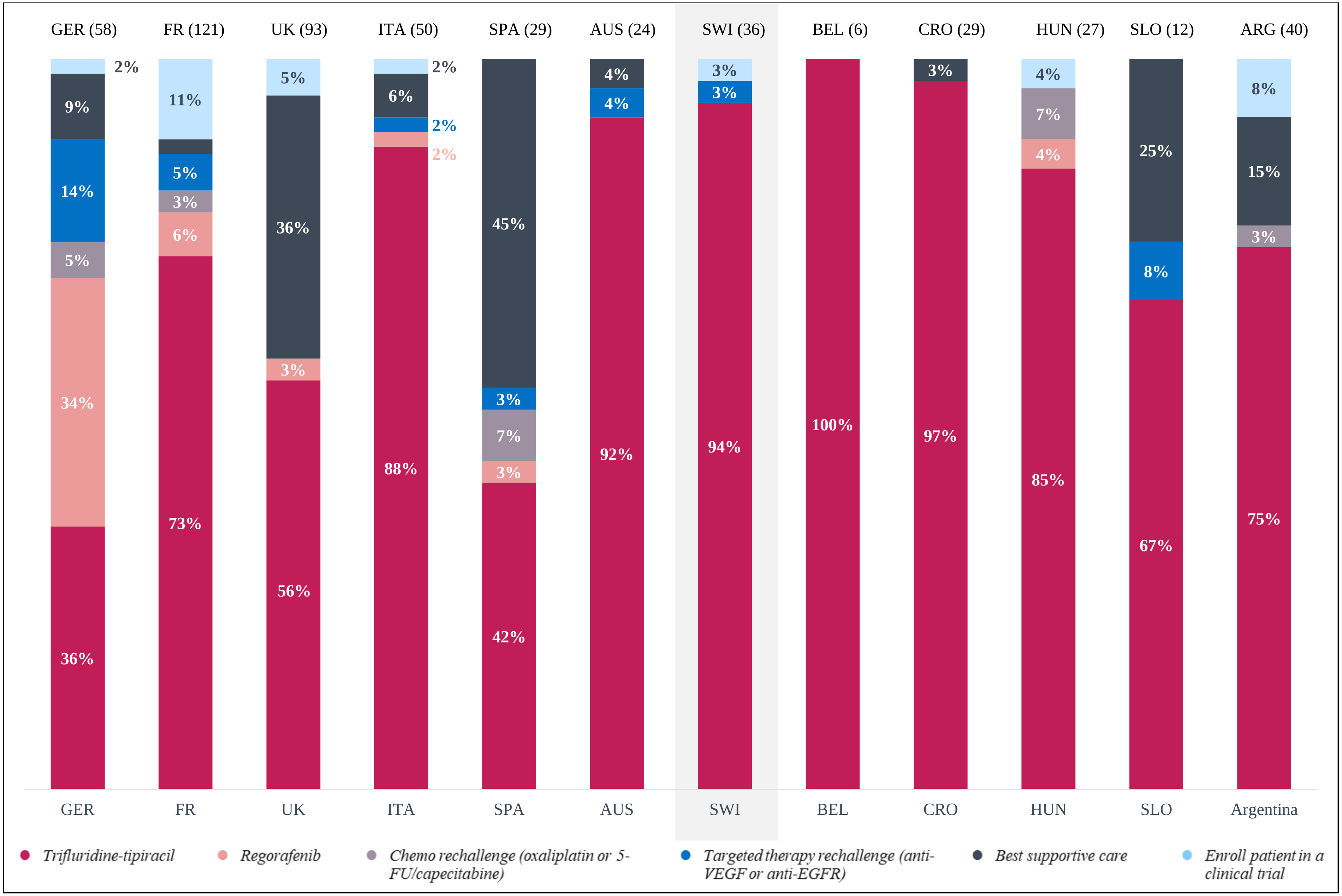

Supplementary Figure S8. Case 3: Preferred Treatment Choice as 3L per Country. Participants were questioned on their preferred third-line treatment choice. Shown as percentage of respondents among participating countries.
