# Supplementary material for: The Screening and COnsensus Based on Practices and Evidence (SCOPE) Program Results of a Survey on Daily Practice Patterns for Patients with Metastatic Colorectal Cancer—A Swiss Perspective in the Context of an International Viewpoint
Source: Curr Oncol. 2022 Aug 6;29(8):5604–15. doi: 10.3390/curroncol29080442 (PMC9406863; doi:10.3390/curroncol29080442)
Supplement: Supplementary file 1 [file curroncol-29-00442-s001.zip › Table S1.pdf]

Supplementary Table S1: Declarative questions of the survey

**Table S1.** Declarative questions of the survey.	
**Question**	**Response options**
What is your primary medical specialty?	Medical oncologist<br>Radio-oncologist<br>Gastroenterologist<br>Surgeon
What type of practice do you mainly work in?	Private office/Private focus office<br>Teaching hospital/University hospital<br>Cancer center/Oncologic reference center<br>General/Non-university public hospital<br>Private hospital/Private clinic
On average, how many patients with metastatic colorectal cancer do you manage in total in a typical month?	<10<br>10–19<br>20–29<br>30–39<br>≥40
How old are you?	<35 years old<br>36–55<br>>55
Which of the following tests do you request for your mCRC patients?[a]	*KRAS* and *NRAS*<br>*KRAS*<br>*BRAF*<br>MSI<br>HER2
In general, what are your treatment goals in first-line mCRC?[b]	Improve progression-free survival<br>Limit toxicity

What are your first-line treatment drivers?[b]	Maintain patient's autonomy<br>Maintain performance status<br>Preserve quality of life<br>Prolong overall survival<br>Relieve symptoms<br>Shrink tumor size<br>Stabilize disease
What are your treatment goals in third-line mCRC?[b]<br><br>What are your third-line treatment drivers?[b]	Improve progression-free survival<br>Limit toxicity<br>Maintain patient's autonomy<br>Maintain performance status<br>Preserve quality of life<br>Prolong overall survival<br>Relieve symptoms<br>Shrink tumor size<br>Stabilize disease

HER2 = human epidermal growth factor receptor 2; mCRC = metastatic colorectal cancer; MSI = microsatellite instability. [a]For each test, the physician had the choice of systematically/in certain patient cases/never or almost never; [b]For each option, physicians were asked to rate them as first, second, or third choice.
