# Supplementary material for: The Screening and COnsensus Based on Practices and Evidence (SCOPE) Program Results of a Survey on Daily Practice Patterns for Patients with Metastatic Colorectal Cancer—A Swiss Perspective in the Context of an International Viewpoint
Source: Curr Oncol. 2022 Aug 6;29(8):5604–15. doi: 10.3390/curroncol29080442 (PMC9406863; doi:10.3390/curroncol29080442)
Supplement: Supplementary file 1 [file curroncol-29-00442-s001.zip › Table S2.pdf]

AGE (YR)	Switzerland	All Countries
<35	15%	28%
35–55	68%	58%
>55	17%	13%
**MEDICAL SPECIALTY**		
Medical oncologist	98%	69%
Radiation oncologist	2%	14%
Gastroenterologist	0%	16%
Surgeon	0%	1%
**THE MAIN TYPE OF PRACTICE SETTING**		
Private office	12%	8%
University hospital	42%	47%
Cancer center	20%	21%
General/Public hospital	24%	18%
Private hospital/clinic	2%	7%
**PATIENTS WITH mCRC PER MONTH**		
40	17%	19%
30–39	5%	10%
20–29	7%	22%
10–19	39%	31%
<10	32%	18%
**ALL COUNTRIES**	**n (%)**	REGIONS
Argentina (AR)	47 (7.5)	**Latin America**
Austria (AUS)	24 (3.8)	**Western Europe**
Belgium (BEL)	6 (1.0)	
France (FR)	130 (20.7)	
Germany (GER)	58 (9.2)	
Italy (ITA)	53 (8.4)	
Spain (SPA)	31 (4.9)	
Switzerland (SWI)	40 (6.5)	
United Kingdom (UK)	125 (19.9)	
Hungary (HUN)	74 (11.8)	**Central/Eastern Europe**
Croatia (CRO)	29 (4.6)	
Slovakia (SLO)	12 (1.9)	

Supplementary Table S2 – **Participant Demographics**. Demographics of Swiss and international participants of SCOPE program between November 2018 and January 2020 (final analysis populations).
